# Supplementary material for: A commensal protozoan attenuates Clostridioides difficile pathogenesis in mice via arginine-ornithine metabolism and host intestinal immune response
Source: Nat Commun. 2024 Apr 2;15:2842. doi: 10.1038/s41467-024-47075-0 (PMC10987486; doi:10.1038/s41467-024-47075-0)
Supplement: Supplementary file 1 — Supplementary Information [file 41467_2024_47075_MOESM1_ESM.pdf]

Supplementary Information

A commensal protozoan attenuates *Clostridioides difficile* pathogenesis in mice through regulating the arginine-ornithine metabolic axis and the host intestinal immune response

Huan Yang, Xiaoxiao Wu, Xiao Li, Wanqing Zang, Zhou Zhou, Yuan Zhou, Wenwen Cui, Yanbo Kou, Liang Wang, Ankang Hu, Lianlian Wu, Zhinan Yin, Quangang Chen, Ying Chen, Zhutao Huang, Yugang Wang*, Bing Gu*

Including: **Supplementary Figures 1-8**

Supplementary data 1

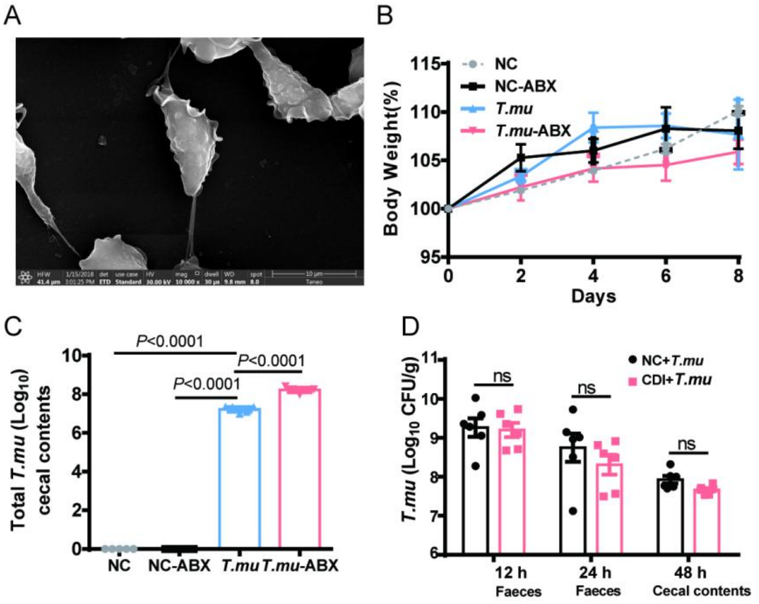

Supplementary Figure 1 | *T.mu* have no obvious influence on normal mice health. (A-C) *T.mu*-negative WT B6 mice were orally administered with purified *T.mu* every two days for a week, along with an antibiotic mixture (ABX) for 5 days. At day 8, the mice were sacrificed to collect the cecal contents. **(A)** Representative SEM image of purified *T.mu*, scale bar: 10 μm. **(B)** Body weight changes in the indicated groups (n = 5 per group). **(C)** The total number of *T.mu* in the indicated groups (n = 5 per group). **(D)** WT B6 mice were colonized with *T.mu* and then infected with *C. difficile*. The fecal and cecal content samples were collected at the indicated time post infection. The number of *T.mu* in the indicated samples were enumerated. NC+*T.mu*: WT mice colonized with *T.mu*; CDI+*T.mu*: WT mice colonized with *T.mu* were infected with *C. difficile* (n = 6 per group). Experiments were repeated independently two times. Data are the mean ± SEM. Statistical significance was determined by two-way ANOVA (**B** and **D**) or one-way ANOVA (**C**). ns: no significance. Source data are provided as a Source Data file.

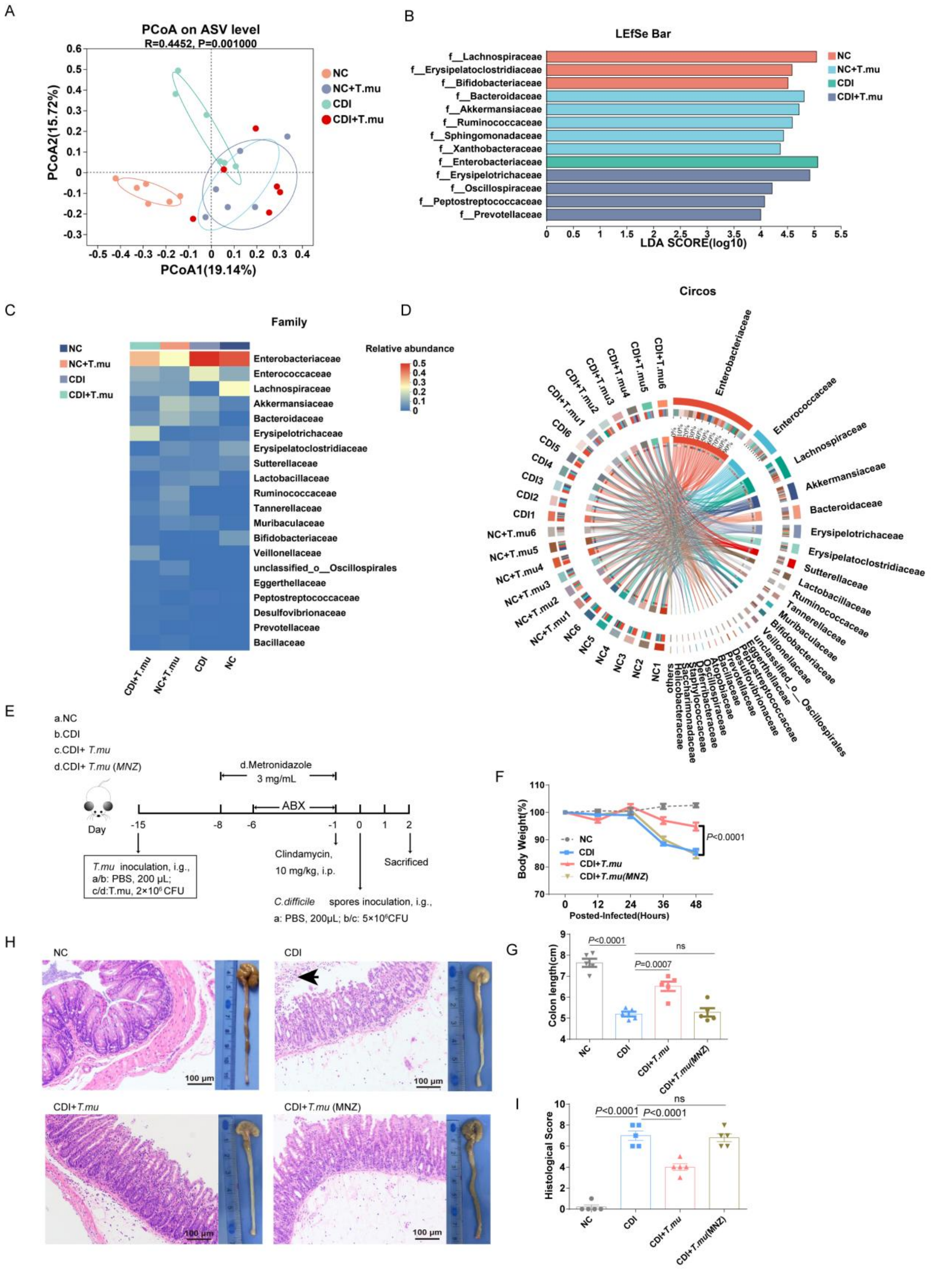

Supplementary Figure 2 | *T.mu's* protective effect on *C. difficile* infection is independent of gut microbiota. (A–D) The cecal contents from the indicated mice were collected at day 2 post infection. Bacterial 16s rRNA sequence analysis was performed on these samples. **(A)** Unweighted principal coordinate analysis (PCoA) conducted on amplicon sequence variant (ASV) abundances presented in the cecal content bacterial community profiles collected from the indicated groups of mice (n = 6 per group). **(B)** Linear discriminant analysis Effect Size (LEfSe) analysis to identify differences in abundant taxa at family phylogenetic level between the indicated

groups (n = 6 per group). **(C)** Heatmap indicating the relative abundance of the top 20 bacterial families presented in the indicated groups of mice 2 days post infection (n = 6 per group). **(D)** Circos plot depicting the correlations between samples and dominant bacterial taxa at family level (n = 6 per group). **(E-I)** Prior to infection, the mice in the CDI+ *T.mu* (MNZ) group were given *T.mu* orally for a week, followed by drinking a 3 mg/ml metronidazole solution for a week to eliminate the *T.mu*. On day 0, each mouse in the CDI and CDI+ *T.mu* (MNZ) groups were inoculated with *C. difficile* spores. The cecum and colon from the indicated mice were collected at day 2 post infection. **(E)** Schematic diagram of *T.mu* elimination experiment design. **(F)** Body weight changes post infection (n = 5 per group). **(G)** Measurement of colon length (n = 5 per group). **(H)** Representative HE-stained images of cecal tissue sections from the indicated mice. Scale bar: 100 μm. Arrow indicates infiltration of inflammatory cells. **(I)** Histological score for HE-stained cecal tissues (n = 5 per group). Experiments were repeated independently two times. Data are the mean ± SEM. Statistical significance was determined by two-way ANOVA (**F**) or one-way ANOVA (**G** and **I**). ns：no significance. Source data are provided as a Source Data file.

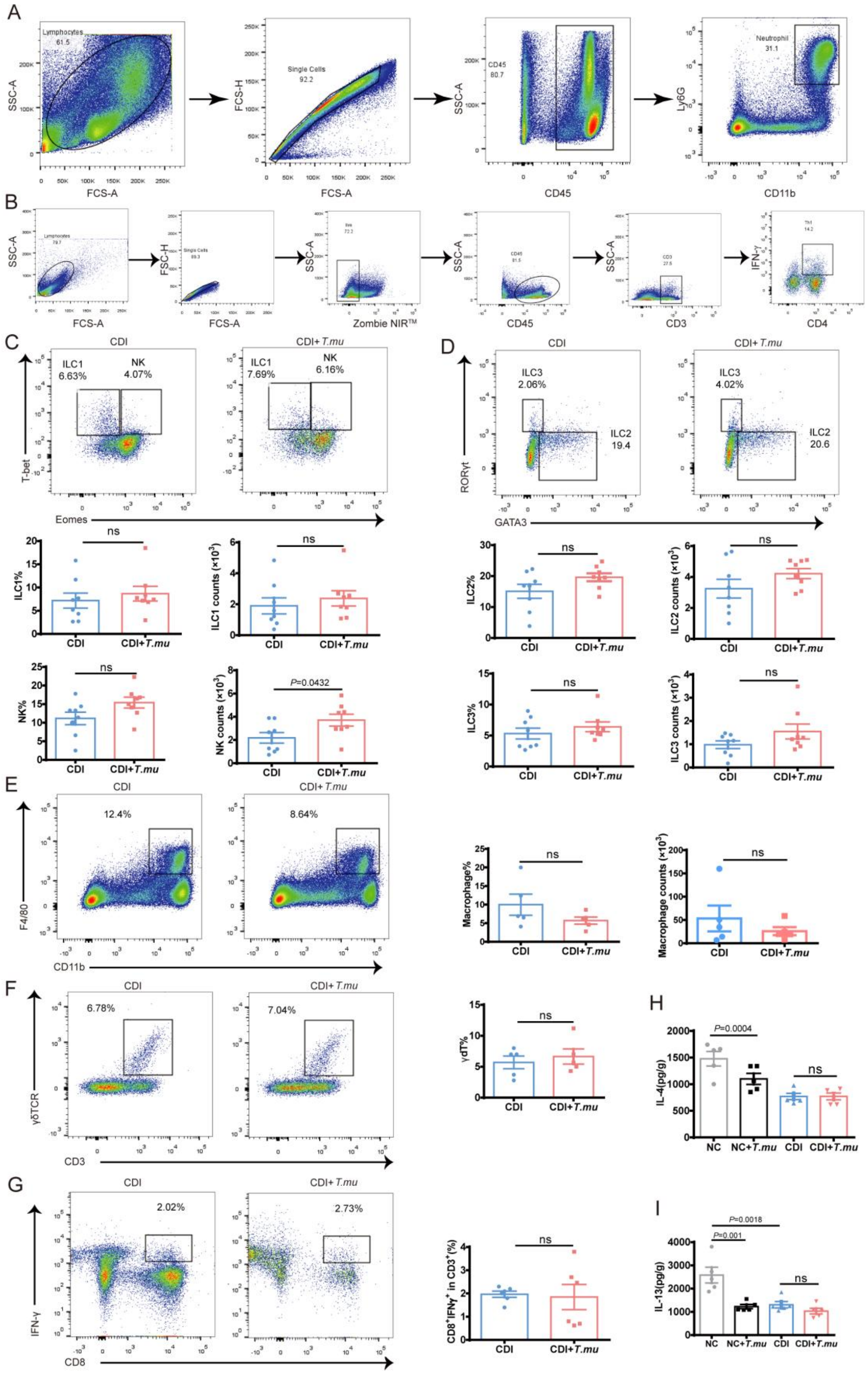

Supplementary Figure 3 | *T.mu* had no influence on ILC, NK and macrophages. The colon from the indicated mice were collected at day 2 post infection. Colonic lamina propria lymphocytes (LPLs) were incubated in the presence of brefeldin A, ionomycin, and PMA for 5 hours and then stained with anti-CD45, CD3, CD8 and IFNγ (Tc1). Colonic LPLs were stained

of NK cells ($CD45^+$ $CD11c^-$ $Gr1^-$ $F4/80^-$ TCR γ/δ^- Fc $\varepsilon R1\alpha^-$ $CD4^-$ $NKp46^+$ ROR gamma-T^- T-bet^+ $EOMES^+$), ILC1 cells ($CD45^+$ $CD11c^-$ $Gr1^-$ $F4/80^-$ TCR γ/δ^- Fc $\varepsilon R1\alpha^-$ $CD4^-$ $NKp46^+$ ROR gamma-T^- T-bet^+ $EOMES^-$), ILC2 cells ($CD45^+$ $CD11c^-$ $Gr1^-$ $F4/80^-$ TCR γ/δ^- Fc $\varepsilon R1\alpha^-$ $CD4^-$ $CD127^+$ $GATA3^+$ ROR gamma-T^-) , ILC3 cells ($CD45^+$ $CD11c^-$ $Gr1^-$ $F4/80^-$ TCR γ/δ^- Fc $\varepsilon R1\alpha^-$ $CD4^-$ $CD127^+$ $GATA3^-$ ROR gamma-T^+). **(A)** Gating strategy for analysis of neutrophil from mice colon LPLs. **(B)** Gating strategy for analysis of Th1 cells from mice colon LPLs. **(C)** Representative flow cytometry graphs and the numbers and percentages of ILC1 and NK cells in colonic LPLs (n = 8 per group). **(D)** Representative flow cytometry graphs and the numbers and percentages of ILC2 and ILC3 cells in colonic LPLs (n = 8 per group). **(E)** Representative flow cytometry graphs and the numbers and percentages of macrophages in colonic LPLs (n = 5 per group). **(F)** Representative flow cytometry graphs and the numbers and percentages of the γδ T cells in colonic lamina propria (n = 5 per group). **(G)** Representative flow cytometry graphs and the numbers and percentages of Tc1 cells in colonic LPLs (n = 6 per group). (**H** and **I**) The IL-4 **(H)** and IL-13 **(I)** levels in the cecum were determined by ELISA (n = 5 per group). Experiments were repeated independently two or three times. Data are the mean ± SEM. Statistical significance was determined by two-sided Students' t test (**C-G**) or one-way ANOVA (**H** and **I**). ns: no significance. Source data are provided as a Source Data file.

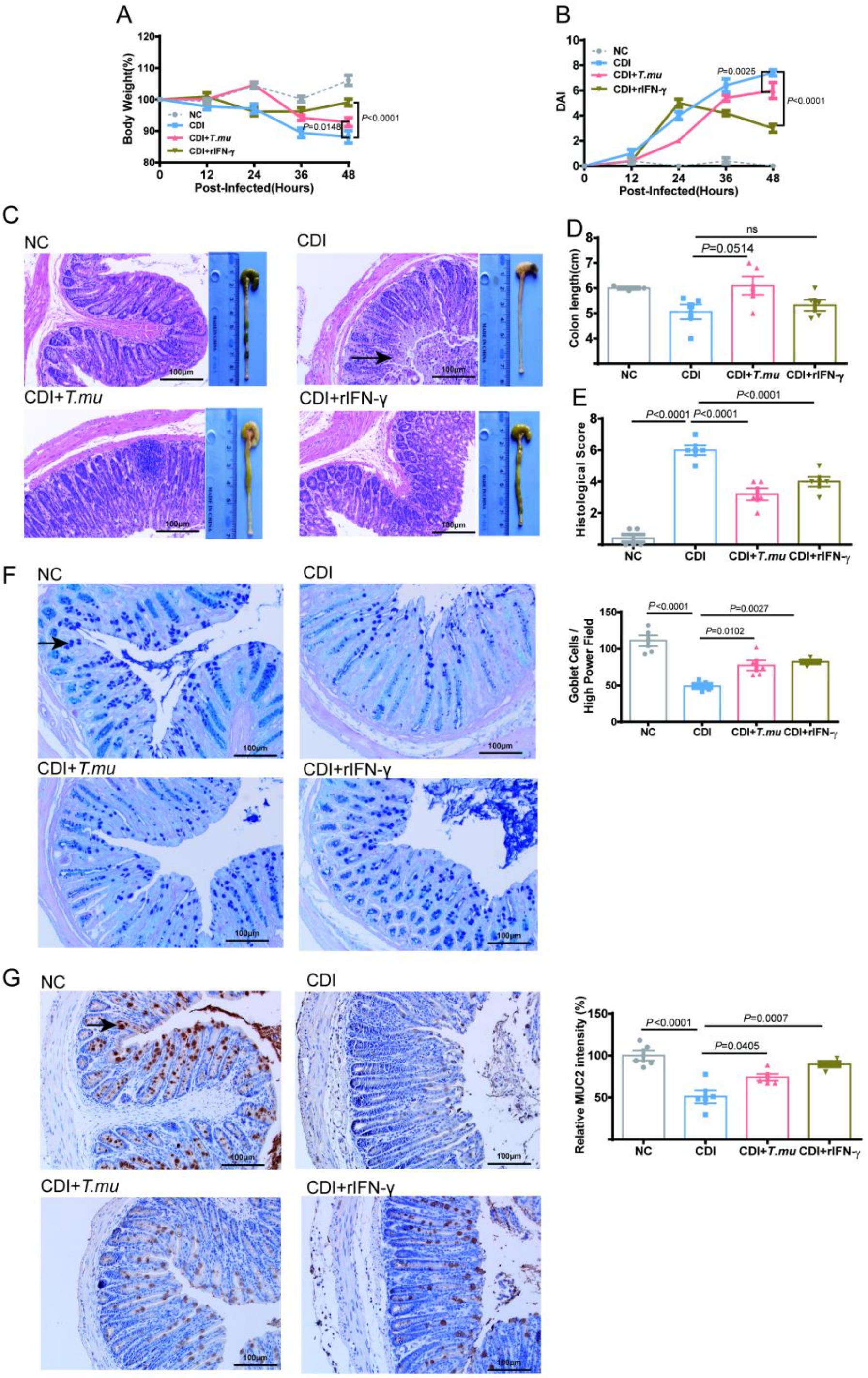

Supplementary Figure 4 | IFN-γ protects intestinal mucosa in CDI mice. (**A-G**) From days -7 to -1, the mice in the CDI+*T.mu* group were administrated with *T.mu* every other day for a total of four times. Each mouse in the CDI+ rIFN-γ group was intraperitoneally injected with 10 μg IFN-γ recombinant protein at 2 h and 24 hours after *C. difficile* infection. On day 0, each mouse in the CDI, CDI+*T.mu*, and CDI+ rIFN-γ groups was inoculated with *C. difficile* spores. The cecum and colon from the mice were collected at day 2 post infection. **(A)** Body weight changes post

infection (n = 5 per group). **(B)** DAI (n = 5 per group). **(C)** Macroscopic photos of colon and representative HE-stained images of colon tissue sections. Scale bar: 100 μm. Arrow indicates infiltration of inflammatory cells. **(D)** Measurement of colon length (n = 5 per group). **(E)** Histological score for HE-stained colon tissues (n = 5 per group). **(F)** Representative PAS- stained images of colon and the goblet cell numbers were enumerated. Scale bar: 100 μm. Arrow indicates goblet cells (n = 5 per group). **(G)** Representative MUC2 histochemical staining images in the colon tissue sections and the staining intensity of MUC2. Scale bar: 100 μm. Arrow indicates MUC2 (n = 5 per group). Experiments were repeated independently two times. Data are the mean ± SEM. Statistical significance was determined by two-way ANOVA (**A** and **B**) or one-way ANOVA (**D-G**), ns: no significance. Source data are provided as a Source Data file.

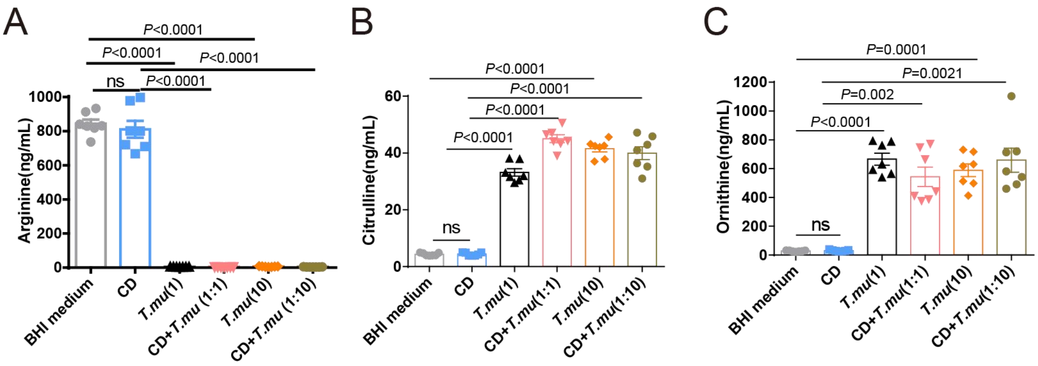

Supplementary Figure 5 | Effects of *T.mu* on arginine-related metabolites in *vitro*. (A-C) After 24 hours of co-culture of 1×10^5 CFU/mL *C. difficile* with or without 1×10^5 or 1×10^6 /mL *T.mu,* respectively. **(A-C)** The level of **(A)** arginine, **(B)** citrulline, and **(C)** ornithine in the Brain Heart Infusion (BHI medium) (n = 7 per group). Experiments were repeated independently three times. Data are the mean ± SEM. Statistical significance was determined by one-way ANOVA (**A-C**), ns: no significance. Source data are provided as a Source Data file.

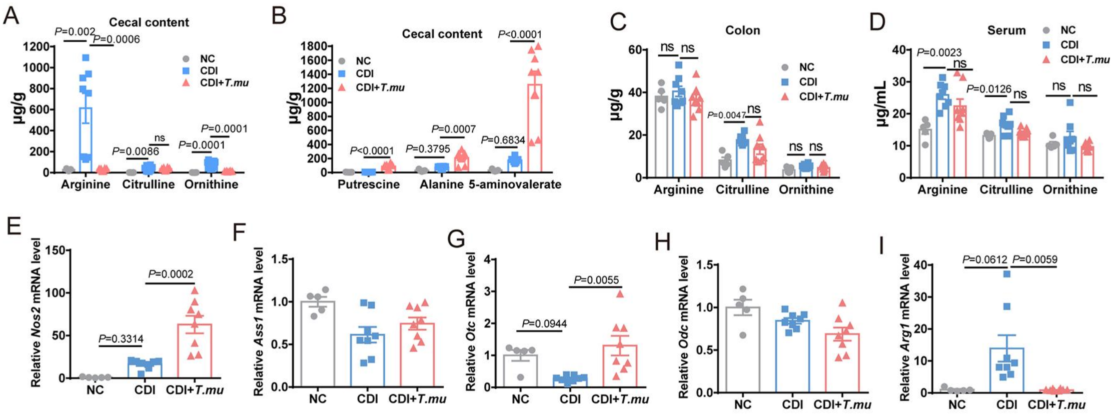

Supplementary Figure 6 | Effects of *T.mu* on intestinal arginine metabolism in germ-free mice. Germ-free B6 mice were divided into 3 groups: normal control group (NC), *C. difficile*-infected group (CDI) and *T.mu* plus *C. difficile* group (CDI+*T.mu*). On days -7, mice in the CDI+*T.mu* group were gavaged orally with purified *T.mu*. On day 0, each mouse in the CDI group and CDI+*T.mu* group was given *C. difficile* spores. Mice were sacrificed at 36 hours after infection. **(A)** The levels of arginine, citrulline, and ornithine in the cecal content at 36 hours after *C. difficile* infection (n = 5 in the NC group, n = 8 in the CDI and CDI+*T.mu* group). **(B)** The levels of putrescine, D-alanine, and 5-aminovalerate in the cecal content at 36 hours after *C. difficile* infection (n = 5-8 per group). (**C** and **D**) The levels of arginine, citrulline, and ornithine in the **(C)** colon and **(D)** serum at 36 hours after *C. difficile* infection (n = 5 in the NC group, n = 8 in the CDI and CDI+T.mu group). **(E-I)** The relative expression levels of **(E)** *Nos2*, **(F)** *Ass1*, **(G)** *Otc*, **(H)** *Odc* and **(I)** *Arg1* in the colon at 36 hours after *C. difficile* infection were determined by qRT-PCR (n = 5 in the NC group, n = 8 in the CDI and CDI+*T.mu* group). Experiments were repeated independently two times. Data are the mean ± SEM. Statistical significance was determined by one-way ANOVA (**A-I**). ns: no significance. Source data are provided as a Source Data file.

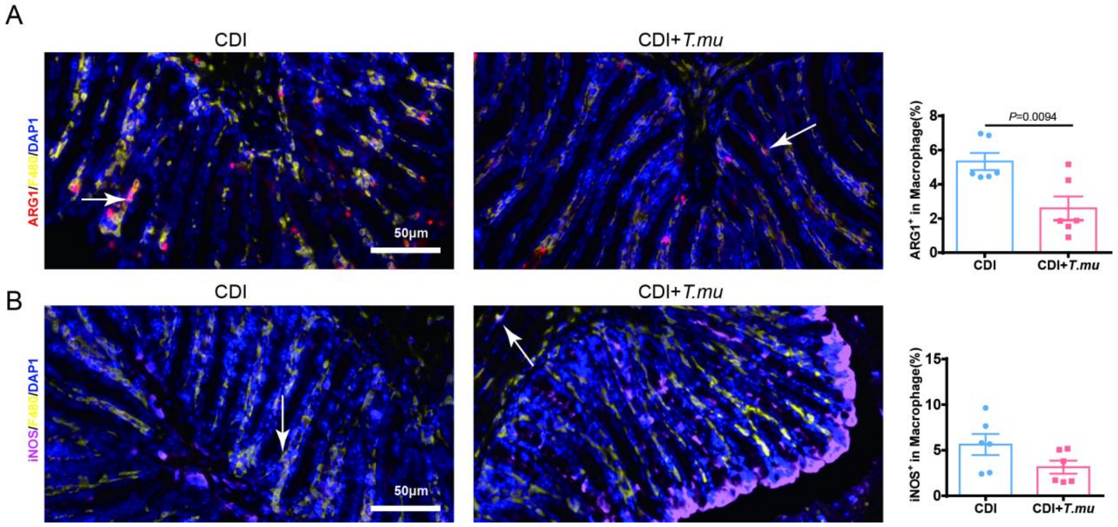

Supplementary Figure 7 | Effect of *T.mu* on ARG1- or iNOS-positive cells in the colon of CDI mice. The colon was collected at day 2 post infection and stained with ARG1 (red), iNOS (purple), F4/80 (yellow), and DAPI (blue). **(A)** Representative immunofluorescence images and the proportion of $ARG1^{+}F4/80^{+}$ macrophages enumerated (n = 6 per group). **(B)** Representative immunofluorescence images and the proportion of $iNOS^{+}F4/80^{+}$ macrophages enumerated. Scale bar, 50 μm (n = 6 per group). The white arrow indicates $ARG1^{+}$ $F4/80^{+}$ macrophages or $iNOS^{+}F4/80^{+}$ macrophages. Experiments were repeated independently two times. Data are the mean ± SEM. Statistical significance was determined by two-sided Students' t test **(A-B)**. Source data are provided as a Source Data file.

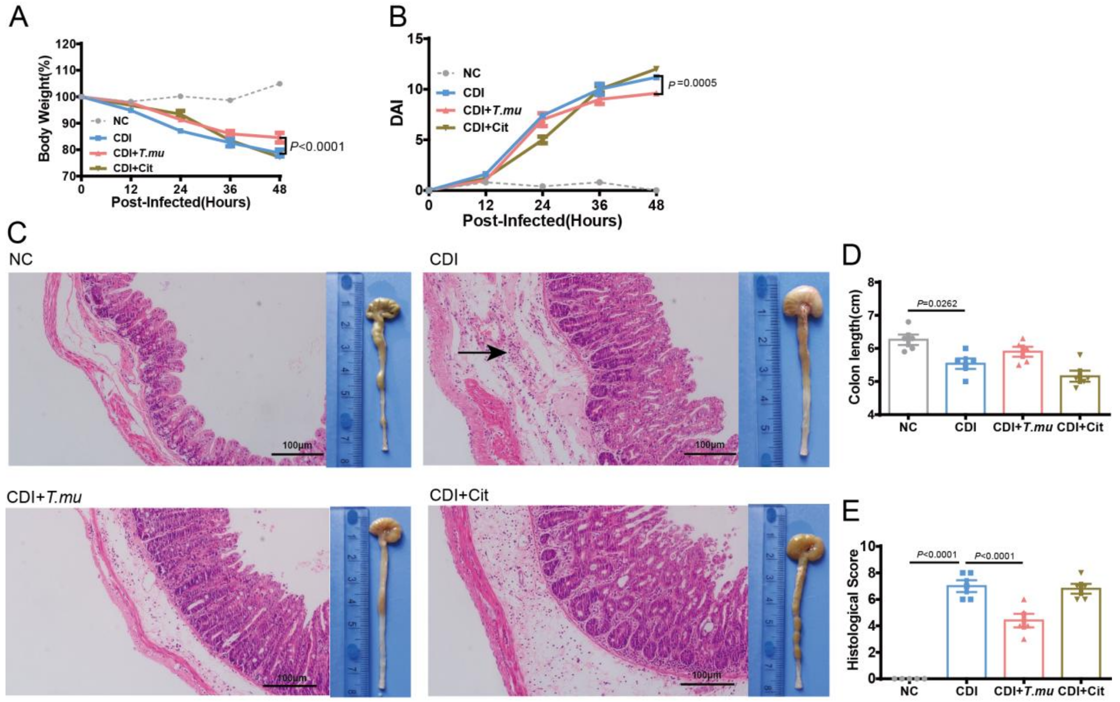

Supplementary Figure 8 | *T.mu* protection is independent of citrulline. (A-E) From days -7 to -1, the mice in the CDI+*T.mu* group were administrated with *T.mu* every other day for a total of four times. Mice in the CDI +Cit group were given 2% citrulline solution in the drinking water for 7 days before infection. On day 0, each mouse in the CDI, CDI+*T.mu*, and CDI+Cit groups was inoculated with *C. difficile* spores. The cecum and colon of the mice were collected at day 2 post infection. **(A)** Body weight changes post infection (n = 5 per group). **(B)** DAI (n = 5 per group). **(C)** Macroscopic photos of colon and representative HE-stained images of the cecal tissue sections. Scale bar: 100 μm. Arrow indicates infiltration of inflammatory cells. **(D)** Measurement of colon length (n = 5 per group). **(E)** Histological score for HE-stained cecal tissues (n = 5 per group). Experiments were repeated independently two times. Data are the mean ± SEM. Statistical significance was determined by two-way ANOVA (**A** and **B**) or one-way ANOVA (**D** and **E**). Source data are provided as a Source Data file.
