# Supplementary material for: A commensal protozoan attenuates Clostridioides difficile pathogenesis in mice via arginine-ornithine metabolism and host intestinal immune response
Source: Nat Commun. 2024 Apr 2;15:2842. doi: 10.1038/s41467-024-47075-0 (PMC10987486; doi:10.1038/s41467-024-47075-0)
Supplement: Supplementary file 3 — Description of Additional Supplementary Files [file 41467_2024_47075_MOESM3_ESM.pdf]

Description of Additional Supplementary Files

Supplementary Data 1: A list of the primers used.
