# Supplementary material for: A commensal protozoan attenuates Clostridioides difficile pathogenesis in mice via arginine-ornithine metabolism and host intestinal immune response
Source: Nat Commun. 2024 Apr 2;15:2842. doi: 10.1038/s41467-024-47075-0 (PMC10987486; doi:10.1038/s41467-024-47075-0)
Supplement: Supplementary file 5 — Reporting Summary [file 41467_2024_47075_MOESM5_ESM.pdf]

nature portfolio

Corresponding author(s): Yugang Wang, Bing Gu

Last updated by author(s): Feb 23, 2024

Reporting Summary

Nature Portfolio wishes to improve the reproducibility of the work that we publish. This form provides structure for consistency and transparency in reporting. For further information on Nature Portfolio policies, see our Editorial Policies and the Editorial Policy Checklist.

Statistics

For all statistical analyses, confirm that the following items are present in the figure legend, table legend, main text, or Methods section.

n/a	Confirmed	
☐	☒	The exact sample size (*n*) for each experimental group/condition, given as a discrete number and unit of measurement
☐	☒	A statement on whether measurements were taken from distinct samples or whether the same sample was measured repeatedly
☐	☒	The statistical test(s) used AND whether they are one- or two-sided<br>*Only common tests should be described solely by name; describe more complex techniques in the Methods section.*
☐	☒	A description of all covariates tested
☐	☒	A description of any assumptions or corrections, such as tests of normality and adjustment for multiple comparisons
☐	☒	A full description of the statistical parameters including central tendency (e.g. means) or other basic estimates (e.g. regression coefficient) AND variation (e.g. standard deviation) or associated estimates of uncertainty (e.g. confidence intervals)
☐	☒	For null hypothesis testing, the test statistic (e.g. *F*, *t*, *r*) with confidence intervals, effect sizes, degrees of freedom and *P* value noted<br>*Give P values as exact values whenever suitable.*
☒	☐	For Bayesian analysis, information on the choice of priors and Markov chain Monte Carlo settings
☒	☐	For hierarchical and complex designs, identification of the appropriate level for tests and full reporting of outcomes
☐	☒	Estimates of effect sizes (e.g. Cohen's *d*, Pearson's *r*), indicating how they were calculated

Our web collection on statistics for biologists contains articles on many of the points above.

Software and code

Policy information about availability of computer code

Data collection	Untargeted metabolomics analyses:The data were analyzed through the free online platform of Majorbio cloud platform (www.cloud.majorbio.com). Metabolic features detected at least 80% in any set of samples were retained. After filtering, minimum metabolite values were imputed for specific samples in which the metabolite levels fell below the lower limit of quantitation and each metabolic features were normalized by sum. To reduce the errors caused by sample preparation and instrument instability, the response intensity of the sample mass spectrum peaks was normalized by the sum normalization method, and the normalized data matrix was obtained. At the same time, variables with relative standard deviation (RSD) > 30% of QC samples were removed, and log10 logarithmization was performed to obtain the final data matrix for subsequent analysis.<br>Microbiome analysis: The length and concentration of the PCR products were determined by agarose gel electrophoresis. PCR products were mixed in equidensity ratios using the GeneTools Analysis Software (Version 4.03.05.0, SynGene).The library was sequenced on an Miseq PE300/NovaSeq PE250 platform (Shanghai Majorbio Technology Co., Ltd. Shanghai, China). Using fastp software (https://github.com/OpenGene/fastp, version 0.20.0) to quality control of original sequencing sequence, use FLASH software (http://www.cbcb.umd.edu/software/flash, version 1.2.7) splicing. A species taxonomic analysis of ASVs was performed using Naive bayes classifiers from Qiime2 based on the Sliva 16S rRNA gene database (v 138). Bioinformatic analysis of the gut microbiota was carried out using the Majorbio Cloud platform (https://cloud.majorbio.com).
Data analysis	the GeneTools Analysis Software, Excel, Adobe Photoshop CS6,Adobe Illustrator CS6, Graphpad Prism 6

For manuscripts utilizing custom algorithms or software that are central to the research but not yet described in published literature, software must be made available to editors and reviewers. We strongly encourage code deposition in a community repository (e.g. GitHub). See the Nature Portfolio guidelines for submitting code & software for further information.

Data

Policy information about availability of data

All manuscripts must include a data availability statement. This statement should provide the following information, where applicable:

- Accession codes, unique identifiers, or web links for publicly available datasets
- A description of any restrictions on data availability
- For clinical datasets or third party data, please ensure that the statement adheres to our policy

The data generated or analyzed during this study are included in this published article and its supplementary information files. Source data are provided with this paper. The Source data are available in Figshare with the identifier [data DOI. “https://doi.org/10.6084/m9.figshare.22580611”]
The MASS SPEC-BASED METABOLOMICS data generated in this study have been deposited in the MetaboLights database under accession the account number:yanghuan2015@tmu.edu.cn, Password:11232002.20fy [https://www.ebi.ac.uk/metabolights/editor/login].The 16S rRNA-seq data have been deposited in the NCBI Sequence Read Archive database (SRA, PRJNA1077800).

Research involving human participants, their data, or biological material

Policy information about studies with human participants or human data. See also policy information about sex, gender (identity/presentation), and sexual orientation and race, ethnicity and racism.

Reporting on sex and gender	*Use the terms sex (biological attribute) and gender (shaped by social and cultural circumstances) carefully in order to avoid confusing both terms. Indicate if findings apply to only one sex or gender; describe whether sex and gender were considered in study design; whether sex and/or gender was determined based on self-reporting or assigned and methods used. Provide in the source data disaggregated sex and gender data, where this information has been collected, and if consent has been obtained for sharing of individual-level data; provide overall numbers in this Reporting Summary. Please state if this information has not been collected. Report sex- and gender-based analyses where performed, justify reasons for lack of sex- and gender-based analysis.*
Reporting on race, ethnicity, or other socially relevant groupings	*Please specify the socially constructed or socially relevant categorization variable(s) used in your manuscript and explain why they were used. Please note that such variables should not be used as proxies for other socially constructed/relevant variables (for example, race or ethnicity should not be used as a proxy for socioeconomic status). Provide clear definitions of the relevant terms used, how they were provided (by the participants/respondents, the researchers, or third parties), and the method(s) used to classify people into the different categories (e.g. self-report, census or administrative data, social media data, etc.) Please provide details about how you controlled for confounding variables in your analyses.*
Population characteristics	*Describe the covariate-relevant population characteristics of the human research participants (e.g. age, genotypic information, past and current diagnosis and treatment categories). If you filled out the behavioural & social sciences study design questions and have nothing to add here, write "See above."*
Recruitment	*Describe how participants were recruited. Outline any potential self-selection bias or other biases that may be present and how these are likely to impact results.*
Ethics oversight	*Identify the organization(s) that approved the study protocol.*

Note that full information on the approval of the study protocol must also be provided in the manuscript.

Field-specific reporting

Please select the one below that is the best fit for your research. If you are not sure, read the appropriate sections before making your selection.

☒ Life sciences ☐ Behavioural & social sciences ☐ Ecological, evolutionary & environmental sciences

For a reference copy of the document with all sections, see nature.com/documents/nr-reporting-summary-flat.pdf

Life sciences study design

All studies must disclose on these points even when the disclosure is negative.

Sample size	For germ-free animal and conventional mouse experiment a sample size of five to ten per group was used based on availability of mice from litters and controlled for age.
Data exclusions	Outliers were excluded only if experimental mistakes happened
Replication	all experiments were repeated at least 2-3 times in various settings.
Randomization	This study mainly involved animal experiments. When grouping mice, the random grouping principle was strictly followed.

Blinding	This study involved histopathological scoring, the preparation and photographing of pathological specimens, and the scoring followed the double-blind principle.

Behavioural & social sciences study design

All studies must disclose on these points even when the disclosure is negative.

Study description	*Briefly describe the study type including whether data are quantitative, qualitative, or mixed-methods (e.g. qualitative cross-sectional, quantitative experimental, mixed-methods case study).*
Research sample	*State the research sample (e.g. Harvard university undergraduates, villagers in rural India) and provide relevant demographic information (e.g. age, sex) and indicate whether the sample is representative. Provide a rationale for the study sample chosen. For studies involving existing datasets, please describe the dataset and source.*
Sampling strategy	*Describe the sampling procedure (e.g. random, snowball, stratified, convenience). Describe the statistical methods that were used to predetermine sample size OR if no sample-size calculation was performed, describe how sample sizes were chosen and provide a rationale for why these sample sizes are sufficient. For qualitative data, please indicate whether data saturation was considered, and what criteria were used to decide that no further sampling was needed.*
Data collection	*Provide details about the data collection procedure, including the instruments or devices used to record the data (e.g. pen and paper, computer, eye tracker, video or audio equipment) whether anyone was present besides the participant(s) and the researcher, and whether the researcher was blind to experimental condition and/or the study hypothesis during data collection.*
Timing	*Indicate the start and stop dates of data collection. If there is a gap between collection periods, state the dates for each sample cohort.*
Data exclusions	*If no data were excluded from the analyses, state so OR if data were excluded, provide the exact number of exclusions and the rationale behind them, indicating whether exclusion criteria were pre-established.*
Non-participation	*State how many participants dropped out/declined participation and the reason(s) given OR provide response rate OR state that no participants dropped out/declined participation.*
Randomization	*If participants were not allocated into experimental groups, state so OR describe how participants were allocated to groups, and if allocation was not random, describe how covariates were controlled.*

Ecological, evolutionary & environmental sciences study design

All studies must disclose on these points even when the disclosure is negative.

Study description	*Briefly describe the study. For quantitative data include treatment factors and interactions, design structure (e.g. factorial, nested, hierarchical), nature and number of experimental units and replicates.*
Research sample	*Describe the research sample (e.g. a group of tagged Passer domesticus, all Stenocereus thurberi within Organ Pipe Cactus National Monument), and provide a rationale for the sample choice. When relevant, describe the organism taxa, source, sex, age range and any manipulations. State what population the sample is meant to represent when applicable. For studies involving existing datasets, describe the data and its source.*
Sampling strategy	*Note the sampling procedure. Describe the statistical methods that were used to predetermine sample size OR if no sample-size calculation was performed, describe how sample sizes were chosen and provide a rationale for why these sample sizes are sufficient.*
Data collection	*Describe the data collection procedure, including who recorded the data and how.*
Timing and spatial scale	*Indicate the start and stop dates of data collection, noting the frequency and periodicity of sampling and providing a rationale for these choices. If there is a gap between collection periods, state the dates for each sample cohort. Specify the spatial scale from which the data are taken*
Data exclusions	*If no data were excluded from the analyses, state so OR if data were excluded, describe the exclusions and the rationale behind them, indicating whether exclusion criteria were pre-established.*
Reproducibility	*Describe the measures taken to verify the reproducibility of experimental findings. For each experiment, note whether any attempts to repeat the experiment failed OR state that all attempts to repeat the experiment were successful.*
Randomization	*Describe how samples/organisms/participants were allocated into groups. If allocation was not random, describe how covariates were controlled. If this is not relevant to your study, explain why.*
Blinding	*Describe the extent of blinding used during data acquisition and analysis. If blinding was not possible, describe why OR explain why blinding was not relevant to your study.*

Did the study involve field work? ☐ Yes ☐ No

Field work, collection and transport

Field conditions	*Describe the study conditions for field work, providing relevant parameters (e.g. temperature, rainfall).*
Location	*State the location of the sampling or experiment, providing relevant parameters (e.g. latitude and longitude, elevation, water depth).*
Access & import/export	*Describe the efforts you have made to access habitats and to collect and import/export your samples in a responsible manner and in compliance with local, national and international laws, noting any permits that were obtained (give the name of the issuing authority, the date of issue, and any identifying information).*
Disturbance	*Describe any disturbance caused by the study and how it was minimized.*

Reporting for specific materials, systems and methods

We require information from authors about some types of materials, experimental systems and methods used in many studies. Here, indicate whether each material, system or method listed is relevant to your study. If you are not sure if a list item applies to your research, read the appropriate section before selecting a response.

Materials & experimental systems

n/a	Involved in the study	
☐	☒	Antibodies
☒	☐	Eukaryotic cell lines
☒	☐	Palaeontology and archaeology
☐	☒	Animals and other organisms
☒	☐	Clinical data
☒	☐	Dual use research of concern
☒	☐	Plants

Methods

n/a	Involved in the study	
☒	☐	ChIP-seq
☐	☒	Flow cytometry
☒	☐	MRI-based neuroimaging

Antibodies

Antibodies used

anti-MUC2 antibody (27675-1-AP, Proteintech, USA); horseradish peroxidase (HRP)-labeled secondary antibody (GB23303, Servicebio);the anti-arginase antibody (ab233548, EPR22033-369, abcam, England) and anti-ASS1 antibody (ab170952, EPR12398, abcam) ; anti-iNOS antibody (ab283655, RM1017, abcam) ;F4/80 antibody (ab300421, EPR26545-166, abcam) ; secondary antibody (PV-6001, ZSGB-BIO, Beijing, China); anti-CD45 conjugated to PE-Cyanine7 (147704, I3/2.3, Biolegend, USA), anti-CD11b conjugated to AlexPacific Blue (101224, M1/70, Biolegend), anti-Ly6G conjugated to PE (127607, 1A8, Biolegend), and anti-F4/80 conjugated to FITC (123108, BM8, Biolegend),anti-CD3 conjugated to PerCP/Cyanine5.5 (100328, 145-2C11, Biolegend), anti-CD4 conjugated to FITC (100510, RM4-5, Biolegend), anti-CD8 conjugated to APC (100711, 53-6.7, Biolegend) ,anti-IFN-γ conjugated to PE (505808, XMG1.2, Biolegend), anti-IL-17A conjugated to Brilliant Violet 421 (506925, TC11-18H10.1, Biolegend), anti-lineage (CD45R, CD11c, Gr-1, TCR b chain, TCR g/d, Fc eR1a, CD4, F4/80) conjugated to FITC (103205, 117305, 108405, 109205, 118105, 134305, 100510, 123108, Biolegend, 1:100 dilution), anti-CD45 conjugated to APC/Fire 750 (103153, 30-F11, Biolegend), anti-CD335 (NKp46) conjugated to Brilliant Violet 711 (137621, 29A1.4, Biolegend), anti-CD127 (IL-7Ra) conjugated to PE/Cyanine7 (135013, A7R34, Biolegendn),anti-T-bet conjugated to Brilliant Violet 605 (644817, 4B10, Biolegend), anti-GATA3 conjugated to PE (653803, 16E10A23, Biolegend), anti-EOMES conjugated to Alexa Fluor 647 (157703, W17001A, Biolegend), anti-ROR gamma (t) conjugated to PerCP-eFluor 710 (46-6981-80, B2D, eBioscience)

Validation

Commercially available immunohistochemistry, immunofluorescence and flow staining antibodies were selected based on their antigen specificity andsuggested application as described on the manufacturer's website and data sheets:
anti-MUC2 antibody (27675-1-AP, Proteintech, USA, 1:1000), https://www.ptglab.com/products/MUC2-Antibody-27675-1-AP.
horseradish peroxidase (HRP)-labeled secondary antibody (GB23303, Servicebio, 1:200), https://www.servicebio.cn/goodsdetail?id=266.
anti-arginase antibody (ab233548, abcam, England, 1:1000) :https://www.abcam.cn/products/primary-antibodies/liver-arginase-antibody-epr22033-369-ab233548.
anti-ASS1 antibody (ab170952, abcam, 1:1000):https://www.abcam.cn/products/primary-antibodies/ass1-antibody-epr12398-ab170952.
anti-iNOS antibody (ab283655, abcam, 1:200) :https://www.abcam.cn/products/primary-antibodies/inos-antibody-rm1017-ab283655.
anti-F4/80 antibody (ab300421, abcam, 1:10000) :https://www.abcam.cn/products/primary-antibodies/f480-antibody-epr26545-166-ab300421.html
secondary antibody (PV-6001, ZSGB-BIO, Beijing, China):http://www.zsbio.com/product/PV-6001
anti-CD45 conjugated to PE-Cyanine7 (147704, Biolegend, USA, 1:200), https://www.biolegend.com/en-us/products/pe-cyanine7-anti-mouse-cd45-antibody-9794

anti-CD11b conjugated to AlexPacific Blue (101224, Biolegend, 1:200), https://www.biolegend.com/en-us/products/pacific-blue-anti-mouse-human-cd11b-antibody-3863
anti-Ly6G conjugated to PE (127607, Biolegend, 1:200), https://www.biolegend.com/en-us/products/pe-anti-mouse-ly-6g-antibody-4777
anti-F4/80 conjugated to FITC (123108, Biolegend, 1:200), https://www.biolegend.com/en-us/products/fitc-anti-mouse-f4-80-antibody-4067
anti-CD45 conjugated to PE-Cyanine7 (147704, Biolegend, 1:100), https://www.biolegend.com/en-us/products/pe-cyanine7-anti-mouse-cd45-antibody-9794
anti-CD3 conjugated to PerCP/Cyanine5.5 (100328, Biolegend, 1:100), https://www.biolegend.com/en-us/products/percp-cyanine5-5-anti-mouse-cd3epsilon-antibody-4191
anti-CD4 conjugated to FITC (100510, Biolegend, 1:100), https://www.biolegend.com/en-us/products/fitc-anti-mouse-cd4-antibody-480
anti-CD8 conjugated to APC (100711, Biolegend, 1:100), https://www.biolegend.com/en-us/products/apc-anti-mouse-cd8a-antibody-150
anti-IFN-γ conjugated to PE (163504, Biolegend, 1:100), https://www.biolegend.com/en-us/products/pe-anti-mouse-ifn-gamma-antibody-20614
anti-IL-17A conjugated to Brilliant Violet 421 (506925, Biolegend, 1:100), https://www.biolegend.com/en-us/products/brilliant-violet-421-anti-mouse-il-17a-antibody-7223
anti-CD45R conjugated to FITC（103205, Biolegend, 1:100）, https://www.biolegend.com/en-us/products/fitc-anti-mouse-human-cd45r-b220-antibody-445
anti-CD11c conjugated to FITC（117305, Biolegend, 1:100）, https://www.biolegend.com/en-us/products/fitc-anti-mouse-cd11c-antibody-1815
anti-Gr-1 conjugated to FITC（108405, Biolegend, 1:100）, https://www.biolegend.com/en-us/products/fitc-anti-mouse-ly-6g-ly-6c-gr-1-antibody-458
anti-TCR β chain conjugated to FITC（109205, Biolegend）, https://www.biolegend.com/en-us/products/fitc-anti-mouse-tcr-beta-chain-antibody-270
anti-TCR γ/δ conjugated to FITC（118105, Biolegend, 1:100）, https://www.biolegend.com/en-us/products/fitc-anti-mouse-tcr-gamma-delta-antibody-2420
anti-Fc eR1a conjugated to FITC（134305, Biolegend, 1:100）, https://www.biolegend.com/en-us/products/fitc-anti-mouse-fcepsilonrialpha-antibody-5949
anti-CD4 conjugated to FITC（100510, Biolegend, 1:100）, https://www.biolegend.com/en-us/products/fitc-anti-mouse-cd4-antibody-480
anti-F4/80 conjugated to FITC（123108, Biolegend, 1:100）, https://www.biolegend.com/en-us/products/fitc-anti-mouse-f4-80-antibody-4067
anti-CD45 conjugated to APC/Fire 750 (103153, Biolegend, 1:100), https://www.biolegend.com/en-us/products/apc-fire-750-anti-mouse-cd45-antibody-13049
anti-CD335 (NKp46) conjugated to Brilliant Violet 711 (137621, Biolegend, 1:100), https://www.biolegend.com/en-us/products/brilliant-violet-711-anti-mouse-cd335-nkp46-antibody-9577
anti-CD127 (IL-7Rα) conjugated to PE/Cyanine7 (135013, Biolegend, 1:100), https://www.biolegend.com/en-us/products/pe-cyanine7-anti-mouse-cd127-il-7ralpha-antibody-6192
anti-T-bet conjugated to Brilliant Violet 605 (644817, Biolegend, 1:20), https://www.biolegend.com/en-us/products/brilliant-violet-605-anti-t-bet-antibody-7907
anti-GATA3 conjugated to PE (653803, Biolegend, 1:20), https://www.biolegend.com/en-us/products/pe-anti-gata3-antibody-9076
anti-EOMES conjugated to Alexa Fluor 647 (157703, Biolegend, 1:200), https://www.biolegend.com/en-us/products/alexa-fluor-647-anti-mouse-eomes-antibody-18078
anti-ROR gamma (t) conjugated to PerCP-eFluor 710 (46-6981-80, eBioscience, 1:100), https://www.thermofisher.cn/cn/zh/antibody/product/ROR-gamma-t-Antibody-clone-B2D-Monoclonal/46-6981-80

Eukaryotic cell lines

Policy information about cell lines and Sex and Gender in Research

Cell line source(s)
State the source of each cell line used and the sex of all primary cell lines and cells derived from human participants or vertebrate models.

Authentication
Describe the authentication procedures for each cell line used OR declare that none of the cell lines used were authenticated.

Mycoplasma contamination
Confirm that all cell lines tested negative for mycoplasma contamination OR describe the results of the testing for mycoplasma contamination OR declare that the cell lines were not tested for mycoplasma contamination.

Commonly misidentified lines
(See ICLAC register)
Name any commonly misidentified cell lines used in the study and provide a rationale for their use.

Palaeontology and Archaeology

Specimen provenance
Provide provenance information for specimens and describe permits that were obtained for the work (including the name of the issuing authority, the date of issue, and any identifying information). Permits should encompass collection and, where applicable, export.

Specimen deposition	*Indicate where the specimens have been deposited to permit free access by other researchers.*
Dating methods	*If new dates are provided, describe how they were obtained (e.g. collection, storage, sample pretreatment and measurement), where they were obtained (i.e. lab name), the calibration program and the protocol for quality assurance OR state that no new dates are provided.*

☐ Tick this box to confirm that the raw and calibrated dates are available in the paper or in Supplementary Information.

Ethics oversight	*Identify the organization(s) that approved or provided guidance on the study protocol, OR state that no ethical approval or guidance was required and explain why not.*

Note that full information on the approval of the study protocol must also be provided in the manuscript.

Animals and other research organisms

Policy information about studies involving animals; ARRIVE guidelines recommended for reporting animal research, and Sex and Gender in Research

Laboratory animals	Wild-type male C57BL/6J mice, aged 4-6 weeks, were purchased from Xuzhou Medical University. The male C57BL/6J Ifngr-/- mice , aged 4-6 weeks, were provided by Professor Zhinan Yin of Jinan University (Guangzhou, China). The mice were bred and maintained under specific pathogen-free (SPF) condition. Male C57BL/6J germ-free mice, aged 4-6 weeks, were purchased from and housed under germ-free conditions in GemPharmatech Co., Ltd (Nanjing, China).
Wild animals	No wild animals were used in this study
Reporting on sex	Male mice were used in the study.
Field-collected samples	The study did not involve sample collected from the field.
Ethics oversight	All animal experiments were performed according to the standards in the Guide for the Care and Use of Laboratory Animals published in 2011 (Institute of Laboratory Animal Resources of National Research Council, United States). All mouse studies were evaluated by the Laboratory Animal Ethics Committee of Xuzhou Medical University (IACUC number: 202202A278), Xuzhou, China.

Note that full information on the approval of the study protocol must also be provided in the manuscript.

Clinical data

Policy information about clinical studies

All manuscripts should comply with the ICMJEguidelines for publication of clinical research and a completedCONSORT checklist must be included with all submissions.

Clinical trial registration	*Provide the trial registration number from ClinicalTrials.gov or an equivalent agency.*
Study protocol	*Note where the full trial protocol can be accessed OR if not available, explain why.*
Data collection	*Describe the settings and locales of data collection, noting the time periods of recruitment and data collection.*
Outcomes	*Describe how you pre-defined primary and secondary outcome measures and how you assessed these measures.*

Dual use research of concern

Policy information about dual use research of concern

Hazards

Could the accidental, deliberate or reckless misuse of agents or technologies generated in the work, or the application of information presented in the manuscript, pose a threat to:

No	Yes	
☐	☐	Public health
☐	☐	National security
☐	☐	Crops and/or livestock
☐	☐	Ecosystems
☐	☐	Any other significant area

Experiments of concern

Does the work involve any of these experiments of concern:

No	Yes	
☐	☐	Demonstrate how to render a vaccine ineffective
☐	☐	Confer resistance to therapeutically useful antibiotics or antiviral agents
☐	☐	Enhance the virulence of a pathogen or render a nonpathogen virulent
☐	☐	Increase transmissibility of a pathogen
☐	☐	Alter the host range of a pathogen
☐	☐	Enable evasion of diagnostic/detection modalities
☐	☐	Enable the weaponization of a biological agent or toxin
☐	☐	Any other potentially harmful combination of experiments and agents

Plants

Seed stocks	No plant specimens were used in this study.
Novel plant genotypes	No plant specimens were used in this study.
Authentication	No plant specimens were used in this study.

ChIP-seq

Data deposition

☐ Confirm that both raw and final processed data have been deposited in a public database such as GEO.

☐ Confirm that you have deposited or provided access to graph files (e.g. BED files) for the called peaks.

Data access links<br>*May remain private before publication.*	*For "Initial submission" or "Revised version" documents, provide reviewer access links. For your "Final submission" document, provide a link to the deposited data.*
Files in database submission	*Provide a list of all files available in the database submission.*
Genome browser session<br>(e.g. UCSC)	*Provide a link to an anonymized genome browser session for "Initial submission" and "Revised version" documents only, to enable peer review. Write "no longer applicable" for "Final submission" documents.*

Methodology

Replicates	*Describe the experimental replicates, specifying number, type and replicate agreement.*
Sequencing depth	*Describe the sequencing depth for each experiment, providing the total number of reads, uniquely mapped reads, length of reads and whether they were paired- or single-end.*
Antibodies	*Describe the antibodies used for the ChIP-seq experiments; as applicable, provide supplier name, catalog number, clone name, and lot number.*
Peak calling parameters	*Specify the command line program and parameters used for read mapping and peak calling, including the ChIP, control and index files used.*
Data quality	*Describe the methods used to ensure data quality in full detail, including how many peaks are at FDR 5% and above 5-fold enrichment.*
Software	*Describe the software used to collect and analyze the ChIP-seq data. For custom code that has been deposited into a community repository, provide accession details.*

Flow Cytometry

Plots

Confirm that:

☒ The axis labels state the marker and fluorochrome used (e.g. CD4-FITC).

☒ The axis scales are clearly visible. Include numbers along axes only for bottom left plot of group (a 'group' is an analysis of identical markers).

☒ All plots are contour plots with outliers or pseudocolor plots.

☒ A numerical value for number of cells or percentage (with statistics) is provided.

Methodology

Sample preparation	To isolate lymphocytes from the colonic lamina propria, the colons were opened longitudinally, washed with PBS to remove luminal faeces, and cut into 1 cm pieces followed by shaking in cold PBS and then incubating with cold PBS containing 10 mM EDTA at 200 rpm and 37 °C for 30 min to remove the epithelial cells. Next, the lamina propria tissues were sliced with pieces and digested with the RPMI 1640 medium containing 5% fetal bovine serum (FBS, HY-T1000, ExCell Bio, Uruguay), 100 U/L penicilin, 0.1 mg/mL streptomycin, 1 mg/mL collagenase (11088866001, Sigma-Aldrich, USA), 1 mg/mL hyaluronic acid (935166, Sigma-Aldrich, USA), and 1 μg/mL DNase I (D806930, MACKLIN) at 100 rpm and 37 °C for 1h. After incubation, the digested solution was filtered through a 70-μm cell strainer to obtain single cell suspensions and resuspended in 40% percoll, followed by centrifugation at 670×g for 30 min at 4 °C to perform percoll gradient separation for obtaining lamina propria lymphocytes. The cell pellets were washed with cold PBS and resuspended in PBS containing 2% FBS. Spleens were directly ground into single cell suspensions and disposed with erythrocyte lysis buffer to obtain splenic lymphocytes. Bone marrows were obtained from long bones of sacrificed mice.
Instrument	FACSCanto II Flow Cytometer (BD Biosciences)
Software	FlowJo, Ashland, OR, USA
Cell population abundance	For neutrophils and macrophages analysis, single cell suspensions were stained for 30 min at 4°C in the dark with the following antibodies: anti-CD45 conjugated to PE-Cyanine7 (147704, I3/2.3, Biolegend, USA, 1:200 dilution), anti-CD11b conjugated to AlexPacific Blue (101224, M1/70, Biolegend, 1:200 dilution), anti-Ly6G conjugated to PE (127607, 1A8, Biolegend, 1:200), and anti-F4/80 conjugated to FITC (123108, BM8, Biolegend, 1:200 dilution).<br>For analysis of Th and Tc cells, 2×106 cells were stimulated with cell activation cocktail (1:500 dilution, 423303, Biolegend) for 5 h at 37°C in 5% CO2. After incubation for 5 h, cells were washed with PBS and stained with Zombie NIR Fixable Viability Kit (423105, Biolegend, 1:200 dilution) for 10 min, then stained with anti-CD45 conjugated to PE-Cyanine7 (147704, I3/2.3, Biolegend, 1:100 dilution), anti-CD3 conjugated to PerCP/Cyanine5.5 (100328, 145-2C11, Biolegend, 1:100 dilution), anti-CD4 conjugated to FITC (100510, RM4-5, Biolegend, 1:100 dilution), anti-CD8 conjugated to APC (100711, 53-6.7, Biolegend, 1:100 dilution) for 40 min at 4°C in the dark. Then cells were fixed with cell fixation buffer (420801, Biolegend) for 30 min at 4°C, permeabilized with intracellular staining perm wash buffer (421002, Biolegend) and stained with anti-IFN-γ conjugated to PE (505808, XMG1.2, Biolegend, 1:100 dilution), anti-IL-17A conjugated to Brilliant Violet 421 (506925, TC11-18H10.1, Biolegend, 1:100 dilution).<br>For analysis of innate lymphoid cells (ILCs), 2×106 cells were stained with anti-lineage (CD45R, CD11c, Gr-1, TCR b chain, TCR g/d, Fc eR1a, CD4, F4/80) conjugated to FITC (103205, 117305, 108405, 109205, 118105, 134305, 100510, 123108, Biolegend, 1:100 dilution), anti-CD45 conjugated to APC/Fire 750 (103153, 30-F11, Biolegend, 1:100 dilution), anti-CD335 (NKp46) conjugated to Brilliant Violet 711 (137621, 29A1.4, Biolegend, 1:100 dilution), anti-CD127 (IL-7Ra) conjugated to PE/Cyanine7 (135013, A7R34, Biolegend, 1:100 dilution) for 50 min at 4°C. After staining with surface markers, cells were fixed for 1 h by True Nuclear Fix solution (424401, Biolegend), then stained with anti-T-bet conjugated to Brilliant Violet 605 (644817, 4B10, Biolegend, 1:20 dilution), anti-GATA3 conjugated to PE (653803, 16E10A23, Biolegend, 1:20 dilution), anti-EOMES conjugated to Alexa Fluor 647 (157703, W17001A, Biolegend, 1:200 dilution), anti-ROR gamma (t) conjugated to PerCP-eFluor 710 (46-6981-80, B2D, eBioscience, 1:100 dilution) in the True Nuclear Perm buffer (424401, Biolegend) for 1 h.<br>Flow cytometry was performed on the FACS Canto Ⅱ Flow Cytometer (BD Bioscience, USA), and analyzed by the FlowJo software.
Gating strategy	Neutrophil (CD45+CD11b+Ly6G+), Macrophage (CD45+CD11b+F4/80+),Th1 cells (CD45+CD3+CD4+IFN-γ+), Tc1 cells (CD45 +CD3+CD8+IFN-γ+). NK cells (CD45+ CD11c- Gr1- F4/80- TCR γ/δ- Fc ξR1α- CD4- NKp46+ ROR gamma-T- T-bet+ EOMES+), ILC1 cells (CD45+ CD11c- Gr1- F4/80- TCR γ/δ- Fc ξR1α CD4- NKp46+ ROR gamma-T- T-bet+ EOMES-), ILC2 cells (CD45+ CD11c- Gr1- F4/80- TCR γ/δ- Fc ξR1α- CD4- CD127+ GATA3+ ROR gamma-T-) , ILC3 cells (CD45+ CD11c- Gr1- F4/80- TCR γ/δ- Fc ξR1α- CD4- CD127+ GATA3- ROR gamma-T+).

☒ Tick this box to confirm that a figure exemplifying the gating strategy is provided in the Supplementary Information.

Magnetic resonance imaging

Experimental design

Design type	*Indicate task or resting state; event-related or block design.*

Design specifications | *Specify the number of blocks, trials or experimental units per session and/or subject, and specify the length of each trial or block (if trials are blocked) and interval between trials.*

Behavioral performance measures | *State number and/or type of variables recorded (e.g. correct button press, response time) and what statistics were used to establish that the subjects were performing the task as expected (e.g. mean, range, and/or standard deviation across subjects).*

Acquisition

Imaging type(s) | *Specify: functional, structural, diffusion, perfusion.*

Field strength | *Specify in Tesla*

Sequence & imaging parameters | *Specify the pulse sequence type (gradient echo, spin echo, etc.), imaging type (EPI, spiral, etc.), field of view, matrix size, slice thickness, orientation and TE/TR/flip angle.*

Area of acquisition | *State whether a whole brain scan was used OR define the area of acquisition, describing how the region was determined.*

Diffusion MRI ☐ Used ☐ Not used

Preprocessing

Preprocessing software | *Provide detail on software version and revision number and on specific parameters (model/functions, brain extraction, segmentation, smoothing kernel size, etc.).*

Normalization | *If data were normalized/standardized, describe the approach(es): specify linear or non-linear and define image types used for transformation OR indicate that data were not normalized and explain rationale for lack of normalization.*

Normalization template | *Describe the template used for normalization/transformation, specifying subject space or group standardized space (e.g. original Talairach, MNI305, ICBM152) OR indicate that the data were not normalized.*

Noise and artifact removal | *Describe your procedure(s) for artifact and structured noise removal, specifying motion parameters, tissue signals and physiological signals (heart rate, respiration).*

Volume censoring | *Define your software and/or method and criteria for volume censoring, and state the extent of such censoring.*

Statistical modeling & inference

Model type and settings | *Specify type (mass univariate, multivariate, RSA, predictive, etc.) and describe essential details of the model at the first and second levels (e.g. fixed, random or mixed effects; drift or auto-correlation).*

Effect(s) tested | *Define precise effect in terms of the task or stimulus conditions instead of psychological concepts and indicate whether ANOVA or factorial designs were used.*

Specify type of analysis: ☐ Whole brain ☐ ROI-based ☐ Both

Statistic type for inference | *Specify voxel-wise or cluster-wise and report all relevant parameters for cluster-wise methods.*

(See Eklund et al. 2016)

Correction | *Describe the type of correction and how it is obtained for multiple comparisons (e.g. FWE, FDR, permutation or Monte Carlo).*

Models & analysis

n/a	Involved in the study
☐	☐ Functional and/or effective connectivity
☐	☐ Graph analysis
☐	☐ Multivariate modeling or predictive analysis

Functional and/or effective connectivity | *Report the measures of dependence used and the model details (e.g. Pearson correlation, partial correlation, mutual information).*

Graph analysis | *Report the dependent variable and connectivity measure, specifying weighted graph or binarized graph, subject- or group-level, and the global and/or node summaries used (e.g. clustering coefficient, efficiency, etc.).*

Multivariate modeling and predictive analysis | *Specify independent variables, features extraction and dimension reduction, model, training and evaluation metrics.*
